# Relative Importance of Glaucoma-Referral Indicators in Retinal Images in a Diabetic Retinopathy Screening Programme in Slovenia: A Cross-Sectional Study

**DOI:** 10.3390/medicina59081441

**Published:** 2023-08-09

**Authors:** Barbara Podnar, Tit Albreht, Barbara Cvenkel

**Affiliations:** 1 Faculty of Medicine, University of Ljubljana, 1000 Ljubljana, Slovenia; tit.albreht@nijz.si (T.A.); barbara.cvenkel@gmail.com (B.C.); 2 Department of Ophthalmology, University Medical Centre Ljubljana, 1000 Ljubljana, Slovenia; 3 National Institute of Public Health, 1000 Ljubljana, Slovenia

**Keywords:** glaucoma, screening, diabetic retinopathy screening programme, retinal image, regression analysis

## Abstract

*Background and Objectives*: Glaucoma is a major cause of irreversible visual impairment and blindness, so its timely detection is crucial. Retinal images from diabetic retinopathy screening programmes (DRSP) provide an opportunity to detect undiagnosed glaucoma. Our aim was to find out which retinal image indicators are most suitable for referring DRSP patients for glaucoma assessment and to determine the glaucoma detection potential of Slovenian DRSP. *Materials and Methods*: We reviewed retinal images of patients from the DRSP at the University Medical Centre Ljubljana (November 2019–January 2020, May–August 2020). Patients with at least one indicator and some randomly selected patients without indicators were invited for an eye examination. Suspect glaucoma and glaucoma patients were considered accurately referred. Logistic regression (LOGIT) with patients as statistical units and generalised estimating equation with logistic regression (GEE) with eyes as statistical units were used to determine the referral accuracy of indicators. *Results*: Of the 2230 patients reviewed, 209 patients (10.1%) had at least one indicator on a retinal image of either one eye or both eyes. A total of 149 (129 with at least one indicator and 20 without) attended the eye exam. Seventy-nine (53.0%) were glaucoma negative, 54 (36.2%) suspect glaucoma, and 16 (10.7%) glaucoma positive. Seven glaucoma patients were newly detected. Neuroretinal rim notch predicted glaucoma in all cases. The cup-to-disc ratio was the most important indicator for accurate referral (odds ratio 7.59 (95% CI 3.98–14.47; *p* < 0.001) and remained statistically significant multivariably. Family history of glaucoma also showed an impact (odds ratio 3.06 (95% CI 1.02–9.19; *p* = 0.046) but remained statistically significant only in the LOGIT multivariable model. Other indicators and confounders were not statistically significant in the multivariable models. *Conclusions*: Our results suggest that the neuroretinal rim notch and cup-to-disc ratio are the most important for accurate glaucoma referral from retinal images in DRSP. Approximately half of the glaucoma cases in DRSPs may be undiagnosed.

## 1. Introduction

Glaucoma is one of the most common causes of irreversible visual impairment and blindness [1]. The prevalence of primary open-angle glaucoma (POAG), the most common type of glaucoma, is estimated to be 2.4% worldwide and 2.3% in Europe [2]. It is rare in people under 40 years of age, but the prevalence increases substantially with age, reaching about 10% in people over 70 [3,4,5,6,7]. The number of people affected by POAG is predicted to increase considerably due to population growth and ageing for people aged 40–80 years from 53 million in 2020 to 80 million in 2040 worldwide and from 5.7 million to 6.4 million in Europe, respectively [3].

Five to ten percent of people with glaucoma become blind in one or both eyes [8] and 10.6–13.5% of blindness in Europe is due to glaucoma [1]. Since glaucoma only becomes symptomatic after substantial damage to the retinal nerve fibre layer has already occurred, approximately 50% of patients are undetected [9]. Because of the low prevalence of glaucoma, population screening has not been shown to be cost-effective. Case-finding, opportunistic screening, and targeted screening of subgroups with a known higher prevalence of glaucoma have been proposed as alternatives [10]. These groups include the elderly, patients with myopia, of black ethnicity, subjects with positive glaucoma family history, and possibly diabetes mellitus. Diabetes and glaucoma are thought to have some common pathophysiological mechanisms [11]. Despite discordant evidence of a connection between glaucoma and diabetes, people with diabetes might be at an increased risk of glaucoma [12,13]. Diabetic retinopathy screening programmes can represent an opportunity for both an opportunistic and a risk-group screening for glaucoma. As in many other countries, in Slovenia, there is a national diabetic retinopathy screening programme (DRSP). Although with a primary goal of detecting diabetic retinopathy, retinal images can also be assessed for glaucomatous changes of the optic nerve head.

To our knowledge, few attempts have been made to evaluate the usefulness of optic nerve head indicators for glaucoma in a real-life screening setting. This study was conducted to determine the undiagnosed glaucoma-finding potential of the Slovenian DRSP and to establish, which indicators proved best for referring patients from DRSP to an ophthalmologist due to the risk of glaucoma.

## 2. Materials and Methods

This is a cross-sectional DRSP-population-based study. The study was conducted according to the World Medical Association’s Declaration of Helsinki and was approved by the National ethics committee of the Republic of Slovenia in June 2019 (Nr. 0120-333/2019/3).

### 2.1. Participants and Data Collection

Our study involved patients followed at the national DRSP at the Department of Ophthalmology, University Medical Centre Ljubljana. Two graders (a general ophthalmologist, trained for image grading, and a glaucoma specialist) consecutively reviewed retinal images for the presence of typical glaucomatous optic nerve head changes (indicators; Table 1) in patients who had attended the DRSP from November 2019 to January 2020 and from May 2020 to August 2020. The interruption and a sooner-than-planned termination were due to COVID-19 pandemics.

Prior to the assessment of retinal images, clear standards were established to define each indicator in a descriptive manner using a representative retinal image. To minimise the subjectivity of the CD ratio assessment, the CD ratio was calculated from the cup and optic disc measurements on the retinal image. In case of disagreement about the retinal image indicators, the assessment of the retinal image was based on the consensus of the assessors.

Retinal images on which the indicators were not clearly visible were considered to be of insufficient quality and were excluded from the study. The retinal image was considered to be of sufficient quality if the optic disc, its borders, and the peripapillary area were clearly visible and in focus, and if there were no shadows (from small pupils) or white areas (overexposed photographs) obscuring the optic disc/peripapillary area.

All patients from the DRSP who had at least one of the indicators and additionally some randomly selected patients without indicators (to increase data variability) were invited for a complete eye examination, which consisted of history, autorefractometry (T KR-1 Auto Kerato-refractometer; TOPCON CORPORATION, Tokyo, Japan), subjective refraction with Snellen optotypes, automated perimetry (Octopus 900: G pattern, TOP strategy; Haag-Streit Diagnostics, Bern, Switzerland), ultrasound pachymetry (Pachmate DGH 55; DGH Technology, Inc., Exton, PA, USA), Goldmann applanation tonometry, anterior segment slit lamp examination, gonioscopy with a Volk 4-mirror gonio-lens and dilated-pupil posterior segment slit lamp examination with a Volk 90D lens. Written informed consent was obtained from each patient before the examination. Eye examinations were performed by the general ophthalmologist, who was blinded to the data about the patients’ indicators from the retinal images. In case of any uncertainty or suspicion regarding the diagnosis, a glaucoma specialist was consulted.

At the clinical examination, one of the following diagnoses was made:

(1) No glaucoma: normal optic nerve head (ONH) without glaucomatous visual field defect (GVFD).
(2) Suspect glaucoma:
  - 0.6 ≤ vertical cup/disc ratio (CDR) < 0.8 OR diffuse neuretinal rim (NRR) thinning OR 0.2 ≤ CDR asymmetry < 0.3 OR violated ISNT rule–all without GVFD.
  - GVFD without glaucomatous ONH changes.
(3) Glaucoma:
  - Diffuse NRR thinning with CDR ≥0.8 regardless of visual field (VF).
  - CDR ≥ 0.6 or diffuse NRR thinning (regardless of CDR) with GVFD.
  - CDR asymmetry ≥ 0.3 regardless of VF.
  - CDR asymmetry ≥ 0.2 with GVFD.
  - NRR notch regardless of VF.

CDR asymmetry was used only for equally shaped and sized ONHs of fellow eyes. The ISNT rule was applied only to normally shaped and sized ONHs.

GVFD was defined as a Bebie curve, showing a local defect and a cluster of 3 contiguous points at the 5% level on the corrected probability plot, using the TOP strategy with the G program of the Octopus 900 perimeter, not explained by any other ocular conditions. In patients, who could not satisfactorily complete visual field testing, diagnoses were made on the basis of structural changes following Foster et al.’s suggestion [14].

Based on the clinical examination, patients fulfilling the criteria for suspected glaucoma and glaucoma were considered accurately referred.

### 2.2. Statistical Analysis

Data are presented for categorical variables as frequencies (percentages), for normally distributed continuous variables as means (standard deviations), and for non-normally distributed continuous variables as medians (interquartile ranges). An independent samples t-test was used when comparing the means of numerical variables and Fisher’s exact test when comparing two binary variables. The normality of the data was assessed using skewness and kurtosis and graphically with a histogram. Independent variables with skewness and/or kurtosis greater than −/+1 were not considered normally distributed.

Regression modelling was used to examine the relationship between the retinal image indicators of glaucomatous optic nerve head changes and their referral accuracy for glaucoma assessment. Variables tested for a confounding effect were age, gender, diabetes duration, HbA1C, history of arterial hypertension or cardiovascular disease, and family history of glaucoma. To check whether analysing both eyes versus only one eye of a patient can pose any important difference in determining which indicators are important for referral, we analysed data twice: simple logistic regression (LOGIT) was used to analyse data with a patient as a statistical unit. The worse eye presented a patient’s diagnosis; when both eyes had the same diagnosis, we randomly selected one with the help of an online random integer generator. A generalised estimating equation technique with logistic regression (GEE) was used to analyse data with an individual eye as a statistical unit taking into account inter-eye correlation. Odds ratios (OR) and their 95% confidence intervals (CI) are presented for both. The level of statistical significance was set at a value of *p* < 0.05. A Hosmer and Lemeshow test and a Quasi likelihood-under-independence model criterion (QIC) were used to assess the goodness of fit for logistic regression and generalised estimating equation techniques, respectively. Statistical analysis was conducted using SPSS Statistics for Windows, version 27.

## 3. Results

### 3.1. The Selection Process

**Figure 1:**
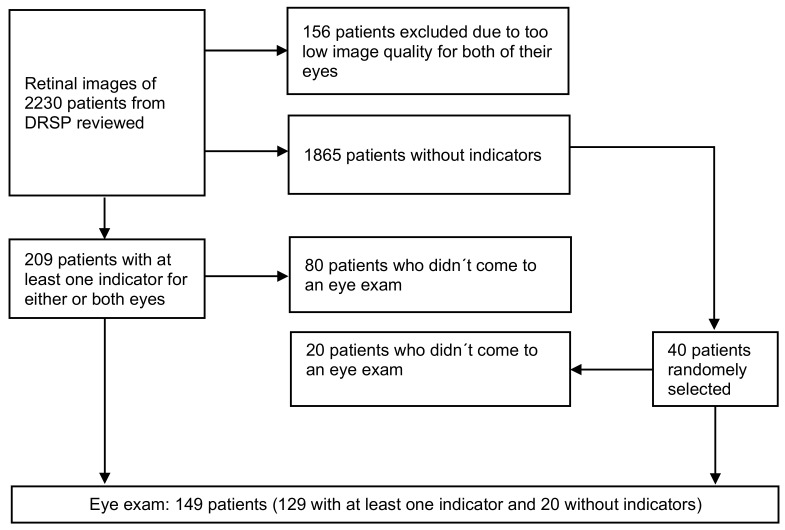
Patient selection process. DRSP, diabetic retinopathy screening programme.

Retinal images of both eyes of 2230 patients were reviewed. Among these, in 156 patients, both eyes were excluded due to inadequate quality of retinal images (opaque ocular media, over-exposed photos, shadows due to small pupils, etc.). Among the rest, there were 209 patients (10.1%) with at least one indicator for either one eye or both eyes. One hundred twenty-nine patients (61.7% of the selected 209) attended the scheduled eye exam. Additionally, we randomly selected 40 patients (1.9%) without indicators. The selection process is shown in Figure 1.

### 3.2. Baseline Characteristics of the Participants

Among the 149 patients who came to the eye exam, there were 86 men and 63 women, with an average age of 69.7 years (SD 10.1) with no statistically significant difference between men and women (*p* = 0.749). The median diabetes duration was 8 years (Table 2). There was no statistically significant difference in age (*p* = 0.166) and gender distribution (*p* = 0.514) between the selected patients who came and those who did not attend the eye exam. The mean intraocular pressure of the patients who came for eye examination was 16.5 (SD 3.2) mmHg for the right eye and 16.4 (SD 3.4) mmHg for the left eye. A total of 32 patients had diabetic retinopathy, 27 of them with at least one indicator and 5 of them without any indicator of glaucomatous ONH changes on retinal images (Table 2).

The most common indicators of glaucomatous ONH changes found on retinal images were irregular NRR and CDR ≥ 0.6 and the rarest were NRR notch and ONH haemorrhage (Table 3).

Among the 129 examined patients with at least one indicator on retinal images sixty-one (47.3%) were diagnosed glaucoma negative, fifty-two (40.3%) were diagnosed suspect glaucoma and sixteen (12.4%) glaucoma positive. Among the 20 examined patients without indicators on retinal images from DRSP, there were 18 diagnosed glaucoma negative and 2 diagnosed suspect glaucoma. Altogether, 70 patients (suspect glaucoma and glaucoma) needed further monitoring for glaucoma onset or progression. Seven (44%) out of a total of sixteen patients with glaucoma were newly detected.

Among the glaucoma suspects were 2 patients (3 eyes) without any described indicator on retinal images. One of them had a cataract and the other had a large optic disc. Among the glaucoma positives were only patients who had at least one indicator on retinal images.

Considering eyes as statistical units, 7 eyes of 7 patients were excluded due to unavailable data about indicators (due to low-quality retinal images), so 291 eyes were available for analysis. Of those, 62.9% were diagnosed glaucoma negative, 28.5% suspect glaucoma, and 8.6% glaucoma positive.

Twenty-seven patients with at least one indicator on retinal images had diabetic retinopathy (DR) (Table 2). Among them, 4 were diagnosed as glaucoma positive, 5 as glaucoma suspect, and 18 as glaucoma negative. A Fisher’s exact test was performed to assess the relationship between the need for referral and the presence of diabetic retinopathy. There was a significant relationship between the two variables, X^2^(1, N = 129) = 5.15, *p* = 0.023—the patients without DR were more likely to need a referral.

### 3.3. Variable Selection for Model Development

Due to unsuitability and unavailability for many eyes (and therefore also for a potential prediction model) and to reduce the selection bias, we excluded CDR asymmetry from model development. Complete data separation was present for NRR notch (i.e., all the cases of NRR notch were in the glaucoma group), so model development using this variable was not possible. For LOGIT, there was only 1 case of ONH haemorrhage so using it as a predictor variable was not reasonable.

### 3.4. Regression Analysis

CDR has been shown to be the strongest indicator for accurate referral. The unadjusted logistic regression model estimated an OR of 7.59 (95% CI 3.98–14.47; *p* < 0.001) for a 0.1 increase in CDR and a similar OR of 5.79 (95% CI 3.42–9.79; *p* < 0.001) when both eyes were analysed and possible inter-ocular correlation taken into account. It remained statistically significant in both multivariable models.

Positive glaucoma family history was also of importance but lost its statistical significance in the multivariable model when both eyes were analysed and inter-ocular correlation was taken into account.

Among the potential confounders female gender and duration of diabetes showed an impact on the referral accuracy when tested univariably but lost their significance in multivariable models.

Complete results of univariable and multivariable logistic regression and generalised estimating equations logistic regression analyses are shown in Table 4 and Table 5, respectively.

## 4. Discussion

The population is ageing and the prevalence of age-related diseases, including glaucoma, is increasing. Since life expectancy is extending, timely detection and treatment of glaucoma is of increasing importance. DRSPs provide an opportunity to detect undiagnosed glaucoma cases. Our study found that 209 out of a total of 2074 patients (10.1%) had optic disc features suspicious for glaucoma on retinal images, which is consistent with the reports of some other studies. Park et al. reported a prevalence of glaucomatous features, defined as rim thinning, nerve fibre defect, or optic disc cupping, found in retinal images of diabetic patients of 10.4% [15]. Cavallerano et al. reported a similar prevalence (9.6%) of large or suspicious optic disc cupping in a telemedicine program for diabetic retinopathy [16].

The average prevalence of glaucoma in Europe was 2.93% in 2013 for the population aged 40–80 years based on a systematic review and meta-analysis [3]; no specific data for Slovenia is available. Our study found a glaucoma prevalence of 0.8% and a suspected glaucoma prevalence of 2.9% in the population of DRSP. The reason for the lower prevalence of glaucoma in DRSP than in the general population could be that DRSP is a highly screened population and many patients, who are found to have glaucomatous optic nerve features and are referred to an ophthalmologist, do not return to DRSP but have their eyes examined for diabetic retinopathy by their ophthalmologist. However, the studies included in the meta-analysis differ in sampling procedures and diagnostic criteria, so no direct comparison is possible. Reported glaucoma and suspect glaucoma prevalence in other DRSPs also varies widely between studies and is difficult to compare due to differences in referral pathways and diagnostic criteria. The lowest prevalence was reported by Steele et al., who used a unique three-step referral pathway with an intermediate step consisting of an eye examination by an optometrist resulting in glaucoma and suspect glaucoma prevalence of 0.25% [17]. Ong et al. reported a prevalence of 0.98%, but the final diagnosis was defined regarding the need for treatment and follow-up of (suspect) glaucoma and not clearly by the structural and/or functional criteria [18]. A study from the USA reported the highest suspect glaucoma prevalence (6.3%) but with rather loose diagnostic criteria based on a CDR equal to or higher than 0.6 and a CDR asymmetry higher than 0.1 with or without GVFD [19]; if GVFD was present, looser structural criteria sufficed for diagnosis. In addition, their sample included 15% African-Americans, in whom glaucomatous optic nerve head changes are more common. The age of the patients studied may also have influenced the suspect glaucoma prevalence, but a comparison of mean ages and glaucoma prevalence in various European DRSP-based studies does not suggest an association between these two factors [17,18,20,21]. The mean age of patients from our study was around 70 years, which is similar to some other studies with different suspect glaucoma prevalences [18,21]. It rather appears that the reported prevalence depends on referral patterns and other-than-age characteristics of the study populations, which were not clearly defined.

About half of patients with glaucoma do not know they have the disease [22]. Our findings are in concordance with this, since among the glaucoma-positive patients, 7 patients (44%) had not previously been diagnosed.

In our study, the presence of NRR notch on a retinal image predicted glaucoma in all cases. Therefore, it could not have been included in the regression analysis. Regarding the relative importance of other indicators included in a multivariable regression model, CDR was the most important variable to predict the accuracy of referral with an OR of 10.88 (95% CI 4.76–24.88; *p* < 0.001) and an OR of 6.18 (95% CI 3.48–10.99; *p* < 0.001) for a 0.1 increase in CDR for LOGIT and GEE analysis, respectively. The big data analysis also showed that CDR was the most important indicator for referral, followed by the NRR notch, for glaucoma specialists and for a deep learning algorithm alike [23].

In our study, besides CDR, only family history of glaucoma retained its statistical significance in a multivariable LOGIT model with an OR of 54.10 (95% CI 3.61–810.34) but with an uncertain estimate of its effect, which decreased when both right and left eye were analysed. The presence of 360° beta PPA was protective in univariable analyses, for which we do not know the explanation. It could be due to sample selection bias; however, it did not remain statistically significant in multivariable analyses. Phene et al. found in a big data analysis mentioned earlier that the presence of beta PPA was the least important in predicting referrable glaucoma among the 11 studied optic nerve head features when the referrable glaucoma was established by glaucoma specialists and second to last when referrable glaucoma was established by the deep learning algorithm—the predictive value of the beta PPA was statistically insignificant in both cases [23]. Interestingly, age was not an important factor in predicting the suspect glaucoma referral, which we believe is due to our sample being composed of selected patients with glaucomatous optic nerve head indicators from the DRSP. Also, none of the other confounders remained statistically significant in a multivariable model. No essential differences in the relative importance of indicators were noted between the analysis with a patient as the statistical unit and the analysis when both patient’s eyes were taken into account, which can be explained by the fact that the most common type of glaucoma is primary open-angle glaucoma, which usually occurs bilaterally, albeit asymmetrically.

The main strength of our study is a thorough ophthalmic examination with clearly defined diagnostic criteria, which served as a reference standard to evaluate the accuracy of referral from DRSP. Since glaucoma cases were easily detected on clinical examination and suspicious glaucoma cases would have required follow-up to establish the definite diagnosis in any case, we decided not to include OCT parameters in our diagnostic criteria.

A strength of our study is also that it is one of the few to evaluate the predictive usefulness of optic nerve head indicators for glaucoma in a real-world setting of a DRSP, resulting in more adequate data on the clinical usefulness of the indicators as compared with other possible study designs.

Our study has some limitations. As it was carried out during the COVID-19-pandemic, some patients, particularly the elderly or with polymorbidities, in whom glaucoma may be more frequent, were afraid of unnecessary medical check-ups, which resulted in a drop-out rate of 40%; there were, however, no differences in age and sex distribution between those attending and those failing to attend the eye examination. The small sample size limits the estimation accuracy of indicators’ effects on the need for referral. This makes it particularly difficult to estimate the impact of rare events, for example, NRR notch or ONH haemorrhage. On the other hand, even a larger sample size cannot even out the differences in the frequencies of the individual indicators which are present in a real-life setting. Nevertheless, the results provide information about which indicators are more important than others and which are of lesser significance. Validation on a larger DRSP sample is needed. Another limitation is that the DRSP is a selected population, and the results may differ in other populations. Also, due to differences in healthcare accessibility or healthcare organisation structure across countries, caution is needed when comparing individual DRSPs.

The indicators were selected according to the literature and were quite broad (covering different aspects of possible glaucomatous optic nerve head changes) to avoid missing the true glaucoma cases. On the other hand, the patients will continue to attend the DR screening programme at least once a year, so changes indicating a suspicious optic disc converting to glaucoma would likely be detected at one of the following visits. The high number of false positive referrals in our study reflects the mentioned relatively broad indicators’ selection on the one hand and a lack of definite criteria for early/suspected glaucoma on the other hand. Only monitoring of borderline cases and finding a change from baseline can confirm that a patient has early glaucoma.

With nearly half of patients undiagnosed, our DRSP has the potential to detect new glaucoma cases. As the other half of the patients already know their diagnosis, it would be useful to ask patients about their glaucoma (or ocular hypertension) history at their DRSP visit to avoid duplication of eye examinations. The same could be considered for a family history of glaucoma, as it is important in predicting suspect glaucoma referrals from the retinal images in DRSP.

In recent years, huge progress has been made in the research of artificial intelligence (AI) and its use in glaucoma screening. Both end-to-end and two-step AI approaches have been used in the studies to predict glaucoma from retinal fundus images [24,25]. However, further research is needed before AI can be routinely used in clinical practice. DRSPs present a valuable source of diverse retinal images to test AI screening performance in the real-world setting.

## 5. Conclusions

In conclusion, this is the first estimation of the prevalence of glaucoma suspects in the DRSP in Slovenia. Since almost half of the cases are undiagnosed, our DRSP has the potential to detect new glaucoma cases. We have shown that the NRR notch and CDR are the most important indicators for accurate glaucoma-referral from retinal images in our DRSP. Further studies on a larger sample are needed to validate this.

## Figures and Tables

**Table 1:** Indicators of glaucomatous optic nerve head changes.

Cup-to-disc ratio ≥ 0.6 * Cup-to-disc ratio asymmetry ≥ 0.2 *
Abnormal neuroretinal rim: diffuse thinning, violated ISNT rule, notches
Optic nerve head haemorrhage
Optic nerve head vascular abnormalities: circumlinear vessels’ baring, collaterals
360° beta peripapillary atrophy

ISNT (inferior-superior-nasal-temporal) rule describes normal neuroretinal rim width, which is broadest inferiorly, followed by superiorly, nasally, and thinnest temporally. * calculated from retinal image cup and disc measurements.

**Table 2:** Baseline characteristics of the participants.

	With at Least One Indicator	Without Indicators	All
Number of participants	129	20	149
Sex			
Male	73	13	86 (57.7%)
Female	56	7	63 (42.3%)
Age, years	70.6	64.7	69.7 (10.1)
Diabetes duration, years	8.0 (4.0; 15.0)	9.0 (4.1; 15.0)	8.0 (4.0; 15.0)
HbA1C, %	7.0	6.9	7.0 (0.9)
AH and/or cardiovascular disease			
No	38	8	46 (30.9%)
Yes	91	12	103 (69.1%)
Family history of glaucoma			
No	115	17	132 (88.6%)
Yes	14	3	17 (11.4%)
Intraocular pressure			
Right eye	16.6 (3.3)	15.5 (2.8)	16.5 (3.2)
Left eye	16.7 (3.5)	14.8 (2.2)	16.4 (3.4)
Diabetic retinopathy			
No	102 (79.1)	15 (75.0)	117 (78.5)
Yes	27 (20.9)	5 (25.0)	32 (21.5)

Data are mean (SD), n (%) or median (interquartile range). HbA1C, haemoglobin A1C levels; AH, arterial hypertension.

**Table 3:** Indicators of glaucomatous optic nerve head changes identified on participants’ retinal fundus images.

Number of Eyes with at Least One Indicator	251
CDR ≥ 0.6	
No	171 (68.1%)
Yes	80 (31.9%)
CDR asymmetry ≥ 0.2 *	
No	84 (86.1%)
Yes	13 (13.4%)
Irregular NRR (violated ISNT rule and/or diffuse NRR thinning) **	
No	143 (58.6%)
Yes	101 (41.4%)
NRR notch	
No	247 (98.4%)
Yes	4 (1.6%)
ONH haemorrhage	
No	245 (97.6%)
Yes	6 (2.4%)
ONH vascular abnormalities	
No	228 (90.8%)
Yes	23 (9.2%)
360° beta PPA	
No	190 (75.7%)
Yes	61 (24.3%)

Data are n (%). CDR, cup-to-disc ratio; NRR, neuroretinal rim; ONH, optic nerve head; PPA, peripapillary atrophy. * data available for 97 patients. ** data available for 244 eyes.

**Table 4:** Predicted OR for a need for referral using logistic regression.

	Univariable OR (95% CI)	*p* Value	Multivariable OR (95% CI)	*p* Value
Age	1.09 (0.94–1.28) per 5 years	0.285	..	..
Sex				
Male	1 (ref)	..	..	..
Female	2.16 (1.11–4.19)	0.023	..	..
Diabetes duration	0.82 (0.69–0.97) per 5 years	0.02	..	..
HbA1C	0.80 (0.56–1.15)	0.231	..	..
AH and/or cardiovascular disease				
No	1 (ref)	..	..	..
Yes	0.84 (0.42–1.68)	0.622	..	..
Family history of glaucoma				
No	1 (ref)	..	..	..
Yes	3.06 (1.02–9.19)	0.046	54.10 (3.61–810.34)	0.004
CDR	7.59 (3.98–14.47) for 0.1 increase	<0.001	10.88 (4.76–24.88) for 0.1 increase	<0.001
CDR asymmetry *	1.30 (0.92–1.83) for 0.1 increase	0.143	/	/
Irregular NRR (violated ISNT rule and/or diffuse NRR thinning)				
No	1 (ref)	..	..	..
Yes	12.12 (5.43–27.05)	<0.001	..	..
ONH vascular abnormalities				
No	1 (ref)	..	..	..
Yes	1.52 (0.53–4.32)	0.434	..	..
360° beta PPA				
No	1 (ref)	.	..	..
Yes	0.16 (0.06–0.42)	<0.001	..	..

145 patients (patient’s worse eye taken as a statistical unit) were analysed using univariable and multivariable analyses of potential factors associated with a need for referral using logistic regression. Parameters with *p* < 0.2 in the univariable analysis were included in the initial multivariable model. Backward stepwise selection was then used to find a model, in which all predictors had *p* < 0.05. This final model included family history of glaucoma and cup-to-disc ratio. OR, odds ratio; CI, confidence interval. * CDR asymmetry not used in the multivariable model (missing values for 35 patients due to differences in size and/or shape of the ONH between the left and right eye of a patient).

**Table 5:** Predicted OR for a need for referral using generalised estimating equations logistic regression.

	Univariable OR (95% CI)	*p* Value	Multivariable OR (95% CI)	*p* Value
Age	1.11 (0.95–1.29) per 5 years	0.141	..	..
Sex				
Male	1 (ref)	..	..	..
Female	1.82 (1.00–3.31)	0.049	..	..
Diabetes duration	0.81 (0.69–0.95) per 5 years	0.01	..	..
				
HbA1C	0.85 (0.60–1.20)	0.355	..	..
AH and/or cardiovascular disease				
No	1 (ref)	..	..	..
Yes	0.91 (0.49–1.72)	0.78	..	..
Family history of glaucoma				
No	1 (ref)	..	..	..
Yes	2.63 (1.07–6.46)	0.035	..	..
CDR	5.79 (3.42–9.79) for 0.1 increase	<0.001	6.18 (3.48–10.99) for 0.1 increase	<0.001
				
CDR asymmetry *	1.32 (1.11–1.56) for 0.1 increase	0.001	/	/
Irregular NRR (violated ISNT rule and/or diffuse NRR thinning)				
No	1 (ref)	..	..	..
Yes	8.35 (4.83–14.42)	<0.001	..	..
ONH haemorrhage				
No	1 (ref)	..	..	..
Yes	1.25 (0.64–2.41)	0.517	..	..
ONH vascular abnormalities				
No	1 (ref)	..	..	..
Yes	1.29 (0.57–2.95)	0.541	..	..
360° beta PPA				
No	1 (ref)	.	..	..
Yes	0.29 (0.15–0.54)	<0.001	..	..

284 eyes of 147 patients were analysed using univariable and multivariable analyses of potential factors associated with a need for referral using generalised estimating equations logistic regression to account for inter-eye correlation. Parameters with *p* < 0.2 in the univariable analysis were included in the initial multivariable model. Backward stepwise selection was then used to find a model, in which all predictors had *p* < 0.05. This final model included only cup-to-disc ratio. * CDR asymmetry not used in the multivariable model (missing values for 64 eyes since values are only possible where retinal images of both eyes of a patient are available and where optic nerve heads of right and left eye are of similar size and shape).

## Data Availability

The data that support the findings of this study are available from the corresponding author, B.P., upon request.
